# Supplementary material for: Complexome profiling of the Chlamydomonas psb28 mutant reveals TEF5 as an early PSII assembly factor
Source: Plant Cell. 2025 Mar 17;37(6):koaf055. doi: 10.1093/plcell/koaf055 (PMC12164590; doi:10.1093/plcell/koaf055)
Supplement: koaf055_Supplementary_Data [file koaf055_supplementary_data.zip › psb28_interactive.zip!/plots/intensity/qualityPlots/peptides/TIM22B_Cre03.g183100.t1.2.html]

### Cre03.g183100.t1.2

Trivial name: TIM22B  
SeqCoverage [median]: 20.80  
Mass: 27.81 kDa  
  
 

| Sequence | MissedCleavage | Mass [Da] | wtIntensity | muIntensity | Score |
| --- | --- | --- | --- | --- | --- |
| AGPWGTAR | 0 | 814.41 | 0.00 | 683.42 | 85.27 |
| LLILHHLDTYR | 0 | 1392.79 | 2662.47 | 17399.81 | 88.68 |
| PANPALDSMMK | 0 | 1173.55 | 19613.74 | 26266.37 | 123.63 |
| YVDNLKK | 1 | 878.49 | 1613.65 | 1956.06 | 104.76 |
|  |  |  | 23889.9 | 46305.7 |  |
